# Characterization of *Salinivibrio socompensis* sp. nov., A New Halophilic Bacterium Isolated from the High-Altitude Hypersaline Lake Socompa, Argentina

**DOI:** 10.3390/microorganisms7080241

**Published:** 2019-08-05

**Authors:** Cristina Galisteo, Cristina Sánchez-Porro, Rafael R. de la Haba, Clara López-Hermoso, Ana B. Fernández, María E. Farias, Antonio Ventosa

**Affiliations:** 1 Department of Microbiology and Parasitology, Faculty of Pharmacy, University of Sevilla, 41012 Sevilla, Spain; 2 Laboratorio de Investigaciones Microbiológicas de Lagunas Andinas, Planta Piloto de Procesos Industriales Microbiológicos, Centro Científico Tecnológico, CONICET Tucumán T4000, Argentina

**Keywords:** *Salinivibrio*, *Salinivibrio socompensis*, bacterial taxonomy, halophilic bacteria, new species, hypersaline lake

## Abstract

The genus *Salinivibrio* belongs to the family *Vibrionaceae* and includes Gram-stain-negative, motile by a polar flagellum, and facultatively anaerobic curved rods. They are halophilic bacteria commonly found in hypersaline aquatic habitats and salted foods. This genus includes five species and two subspecies. A presumed novel species, strain S35^T^, was previously isolated from the high-altitude volcanic, alkaline, and saline lake Socompa (Argentinean Andes). In this study we carried out a complete taxonomic characterization of strain S35^T^, including the 16S rRNA gene sequence and core-genome analysis, the average nucleotide identity (ANIb, ANIm, and orthoANI), and *in silico* DNA–DNA hybridization (GGDC), as well as the phenotypic and chemotaxonomic characterization. It grew at 3%–20% (w/v) NaCl, pH 6–10, and 10–42 °C, with optimum growth at 7.0%–7.5% (w/v) NaCl, pH 8.0, and 37 °C, respectively. Strain S35^T^ was oxidase- and catalase-positive, able to produce acid from D-glucose and other carbohydrates. Hydrolysis of DNA, methyl red test, and nitrate and nitrite reduction were positive. Its main fatty acids were C_16:0_, C_16:1_ *ω*7*c* and C_16:1_ *ω*6*c*, and C_18:1_ *ω*7*c* and/or C_18:1_ *ω*6*c*. ANI, GGDC, and core-genome analysis determined that strain S35^T^ constitutes a novel species of the genus *Salinivibrio*, for which the name *Salinivibrio* *socompensis* sp. nov. is proposed. The type strain is S35^T^ (= CECT 9634^T^ = BNM 0535^T^).

## 1. Introduction

The two main groups that inhabit hypersaline environments are the extremely halophilic archaea (also called haloarchaea) and the moderately halophilic bacteria [1,2]. The haloarchaea are classified within the class *Halobacteria* with three orders: *Haloferacales*, *Halobacteriales*, and *Natrialbales* [3]. Regarding moderately halophilic bacteria, they are very heterogeneous and are represented by a large number of species belonging to at least eight phyla of the domain *Bacteria*: *Proteobacteria*, *Firmicutes*, *Actinobacteria*, *Spirochaetes*, *Bacteroidetes*, *Thermotoga*, *Cyanobacteria*, and *Tenericutes* [2].

Halophilic microorganisms have an important biotechnological potential due to their exceptional physiological and biochemical characteristics. Among their most interesting applications are found the production of a novel restriction enzyme [4], agarases, with industrial and medical applications [5], or β-galactosidase, to produce lactose-free dairy products [6]. Other applications include the production of ectoines, polysaccharides, or extracellular enzymes [1]. 

The genus *Salinivibrio* belongs to the family *Vibrionaceae*, order *Vibrionales*, within the class *Gammaproteobacteria* in the phylum *Proteobacteria* [7]. The first strain isolated was named as *Vibrio costicola* by Smith [8] from bacon rib bones, which was also found lately in aquatic hypersaline environments [9]. *V. costicola* has the ability to grow over a wide range of salt concentrations, and for this reason it has been used as a model microorganism for studies of osmoregulation and other physiological mechanisms [10,11,12,13]. Relevant phenotypic differences and low phylogenetic relationships with other species of the genus *Vibrio*, including *V. costicola*, permitted the reclassification of *V. costicola* in a separate genus, *Salinivibrio* [14]. The genus *Salinivibrio* comprises five species: *Salinivibrio costicola,* with two subspecies: *S. costicola* subsp. *costicola* [8,14,15] and *S. costicola* subsp. *alcaliphilus* [16]; *S. proteolyticus* [17]; *S. siamensis* [18]; *S. sharmensis* [19]; and the recently described *S. kushneri* [20]. *S. costicola* subsp. *vallismortis* [15] was also included as a subspecies of *S. costicola* but according to López-Hermoso et al. [21] it belongs to the species *S. proteolyticus*. The species of this genus are Gram-stain-negative curved rods (0.5–0.6 x 1.0–3.2 µm), motile by one polar flagellum, and non-endospore forming. They are moderately halophilic, able to grow on a NaCl range from 1% to 20% (w/v), with a temperature range from 5 to 50 °C, and pH range from 5 to 10, with the optimal growth being at 2.5% to 10% (w/v) NaCl, 37 °C, and pH 7.3 to 7.5, respectively. Facultatively anaerobic, catalase, and oxidase positive. The DNA G+C content ranges from 49.3 to 52.5 mol% [7,14,16,17,18,19,20].

In 2017, López-Hermoso et al. [22] carried out an extensive revision of the genus *Salinivibrio*, studying 70 strains, including new isolates from different solar salterns, where these bacteria are common habitants, as well as the type strains of the species and subspecies of this genus. Initially, their 16S rRNA gene sequences were compared with those of the known species and subspecies of *Salinivibrio*. A multilocus sequence analysis (MLSA) based on the genes *gyrB, recA, rpoA,* and *rpoD*, validated with DNA–DNA hybridization (DDH) studies, allowed the researchers to classify them into four phylogroups and one phylotype. In 2014, Gorriti et al. [23] reported the draft genome of three strains designated as S34, S35, and S10B. Those strains belonged to the genus *Salinivibrio*; however, in this paper the authors did not perform their systematic characterization, and their taxonomic position was not clarified. These three bacteria were isolated from lake Socompa, at the base of volcano Socompa, located in the Northwestern region of Puna (Argentina) at 3570 m above the sea level. The most relevant features of this lake are high alkalinity (pH 8.5), salinity up to 7% (w/v) NaCl, and extremely high arsenic concentration. It is also remarkable for the presence of actively forming stromatolites [24,25,26], which are considered evidence of early life on Earth [27,28,29]. The aim of this study was to carry out an exhaustive characterization of *Salinivibrio* sp. strain S35^T^, selected as representative of these three new isolates due to the unavailability to cultivate the other two strains. We show that strain S35^T^ constitutes a new species for which we propose the designation of *Salinivibrio socompensis* sp. nov.

## 2. Materials and Methods

### 2.1. Bacterial Strains

The strain S35^T^ was isolated by M. E. Farias, from Laboratorio de Investigaciones Microbiológicas de Lagunas Andinas (LIMLA-PROIMI), Argentina [23]. The type strains of all species of the genus *Salinivibrio,* obtained for culture collections, were included as reference: *Salinivibrio costicola* subsp. *costicola* CECT 4059^T^ [17], *Salinivibrio costicola* subsp. *alcaliphilus* DSM 16359^T^ [19], *Salinivibrio proteolyticus* DSM 19052^T^ [20], *Salinivibrio siamensis* JCM 14472^T^ [21], *Salinivibrio sharmensis* DSM 18182^T^ [22], and *Salinivibrio kushneri* AL184^T^ [23].

### 2.2. Culture Media and Growth Conditions

The strains were routinely grown on SW7.5 medium at 37 °C. The composition of this medium was the following (g l^−1^): NaCl, 58.5; MgCl_2_·6H_2_O, 9.75; MgSO_4_·7H_2_O, 15.25; CaCl_2_, 0.25; KCl, 1.5; NaHCO_3_, 0.05; NaBr, 0.175; and yeast extract, 5.0 [23]. When necessary, the medium was solidified with 2.0% (w/v) agar. The pH was adjusted to 7.5 with 1 M KOH. SW7.5 broth with 20% (v/v) glycerol was used for long-term preservation at −80 °C. For chemotaxonomic characterization, the bacteria were grown for seven days at 37 °C in medium 1, described by Romano et al. [22], containing (g l^−1^): yeast extract, 10.0; NaCl, 100.0; Na_3_-citrate, 3.0; Na_2_CO_3_, 3.0; KCl, 2.0; MgSO_4_·7H_2_O, 1.0; MnCl_2_·4H_2_O, 0.00036; and FeSO_4_, 0.05.

### 2.3. Taxophylogenomic Characterization

#### 2.3.1. Phylogenetic Analysis Based on 16S rRNA Gene Sequence Comparison

**Figure 1:**
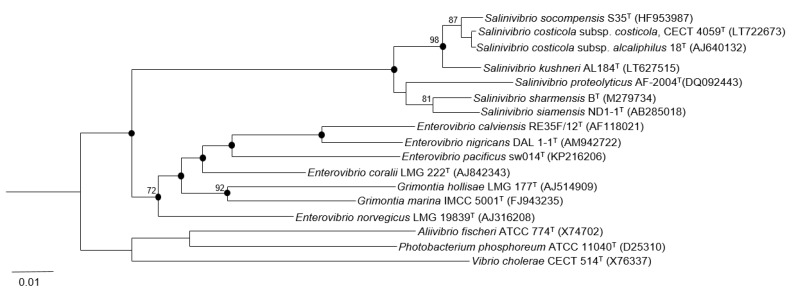
Neighbor-joining phylogenetic tree based on the 16S rRNA gene sequence comparison, showing the relationships between strain S35^T^ and other members of the genus *Salinivibrio* and the family *Vibrionaceae*. Filled circles indicate nodes that were also obtained in trees based on maximum parsimony and maximum likelihood algorithms. Bootstrap values over 70% are shown at the nodes. The accession numbers are shown in parenthesis. Bar, 0.01 substitutions per nucleotide position.

The DNA of strain S35^T^ was obtained using the commercial kit G-spinTM Total DNA Extraction Kit (INtRON Biotechnology), following the instructions of the manufacturer. The 16S rRNA gene was amplified by PCR using the primers 16F27 (5′-AGAGTTTGATCMTGGCTCAG-3′) and 16R1488 (5′-CGGTTACCTTGTTAGGACTTCACC-3′) [30]. The PCR product was purified using the commercial kit MEGAquick-spinTM Plus (INtRON Biotechnology), and sequenced using the Sanger method with oligonucleotides 16F27, 16R1488, 16R343 (5′-ACTGCTGCCTCCCGTA-3′), and 16F530 (5′-GTGCCAGCAGCCGCGG-3′) by StabVida (Oeiras, Portugal). The gene sequences were assembled and edited by ChromasPro software Version 1.5 (Technelysium Pty) and used for initial BLAST, searched against “nt” database in GenBank. To determine the percentages of similarity between strain S35^T^ and the most closely related taxa we used the EzBioCloud.net server [31]. The 16S rRNA gene analysis and phylogenetic trees construction were performed with the ARB software package [32]. Phylogenetic trees were constructed using three different methods: Neighbor-joining [33], maximum parsimony [34], and maximum likelihood [35] algorithms integrated in the ARB software for phylogenetic inference. A bootstrap analysis (1000 replications) was performed to evaluate the robustness of the phylogenetic trees [36]. The 16S rRNA gene sequences of the reference type strains used for the phylogenetic comparison were obtained from GenBank database and their accession numbers are shown in Figure 1.

#### 2.3.2. Phylogenomic Comparative Analysis

For the phylogenomic comparative analysis we used the genomes available from GenBank database that are shown in Table S1. These genomes correspond to the following strains used in this comparative study: *Salinivibrio* sp. strain 35^T^, *S. costicola* subsp. *costicola* LMG 11651^T^, *S. costicola* subsp. *alcaliphilus* DSM 16359^T^, *S. proteolyticus* DSM 19052^T^, *S. siamensis* JCM 14472^T^, *S. sharmensis* DSM 18182^T^, and *S. kushneri* AL184^T^. The quality of these genome sequences is in accordance with the minimal standards for the use of genome data for the taxonomy of prokaryotes [37].

To determine the core genome, the Enveomics [38] tool was used to perform an all-versus-all BLAST search based on nucleotide gene sequences of strain S35^T^ and type strains of the genus *Salinivibrio* to identify clusters of orthologous genes (OGs). Those OGs shared among all taxa and present in single copy per genome were selected. They were aligned with Muscle v. 3.8.31 [39] and subsequently concatenated. An approximately maximum-likelihood tree was constructed using FastTree v. 2.1.9 [40] with the JTT replacement matrix [41] under the CAT approximation (single rate for each site) with 20 rate categories. Local support values were estimated with the Shimodaira-Hasegawa test [42].

#### 2.3.3. Average Nucleotide Identity (ANI) and *n-silico* DNA–DNA Hybridization (DDH)

The average nucleotide identity (ANI) among *Salinivibrio* sp. strain 35^T^ and *S. costicola* subsp. *costicola* LMG 11651^T^, *S. costicola* subsp. *alcaliphilus* DSM 16359^T^, *S. proteolyticus* DSM 19052^T^, *S. siamensis* JCM 14472^T^, *S. sharmensis* DSM 18182^T^, and *S. kushneri* AL184^T^ was calculated using three different methods: The percentages of ANIb based on BLAST+ and ANIm based on MUMmer were performed with JSpeciesWS [43], and the orthoANI was calculated with ChunLab’s Orthologous Average Nucleotide Identity Tool (OAT) [44], available on the EzBioCloud server. *In-silico* DDH was calculated by the bioinformatic tool Genome-to-Genome Distance Calculator (GGDC version 2.1) available from the Leibniz Institute DSMZ [45].

### 2.4. Phenotypic Characterization

#### 2.4.1. Morphology and Motility

Morphology and pigmentation of colonies were observed on SW7.5 solid medium at pH 7.5 after 24 h at 37 °C. Cell morphology and motility were examined by phase-contrast microscopy (Olympus CX41 with DP70 digital camera).

#### 2.4.2. Physiological Characteristics

The range and optimal conditions of salinity for growth were determined by using SW liquid medium at pH 7.5 supplemented with 3%, 6%, 7%, 7.5%, 8%, 9%, 10%, 15%, and 20% (w/v) total salts respectively. In order to determine the optimal (and range) growth at different pH values of strain S35^T^, the isolate was cultured under the optimal salt concentration conditions, adjusting the medium to pH 5, 6, 7, 7.5, 8, 9, and 10, respectively, with appropriate buffers. The optimal and range of temperature were determined by incubating strain S35^T^ under the optimal salt concentration and pH conditions, at temperatures of 4, 10, 15, 28, 37, 40, 42, 45, and 48 °C, respectively. Growth rates were determined by monitoring the increase in the optical density (O.D.) at 600 nm (ThermoSpectronics Spectronic 20D+).

#### 2.4.3. Biochemical Characteristics

Catalase activity was determined by adding 3% (w/v) H_2_O_2_ solution to colonies and observing bubble presence [46]. Oxidase activity was examined using 1% (v/v) tetramethyl-p-phenylenendiamine [47]. Hydrolysis of gelatin, starch, Tween 80, DNA, casein, and aesculin; production of indole, methyl red, and Voges–Proskauer tests; Simmon’s citrate, nitrate, and nitrite reduction; H_2_S production; ornithine, arginine, and lysine decarboxylases; urease; and phenylalanine deaminase were determined as described by Cowan and Steel [46], with the addition of 7.5% (w/v) total salts to the medium [9]. Acid production from carbohydrates was determined using a modified phenol red base medium prepared with SW7.5 supplemented with 1% (w/v) carbohydrate [9,46]. The carbohydrates studied were: D-arabinose, D-fructose, D-galactose, glycerol, D-glucose, lactose, maltose, mannitol, D-sucrose, D-trehalose, and D-xylose.

#### 2.4.4. Nutritional Characteristics

The medium described by Koser [48], as modified by Ventosa et al. [9], was used for the determination of the growth using different carbohydrates, alcohols, organic acids, and amino acids as carbon and energy or carbon, nitrogen, and energy sources. Substrates were added as filter-sterilized solutions to give a final concentration of 1 g L^−1^ for all substrates except for carbohydrates, whose final concentration was 2 g L^−1^. The studied substrates were: amygdalin, D-arabinose, cellobiose, D-fructose, D-galactose, D-glucose, lactose, maltose, mannose, melibiose, melezitose, raffinose, sucrose, starch, D-trehalose, D-xylose, butanol, dulcitol, ethanol, glycerol, mannitol, methanol, propranolol, sorbitol, xylitol, acetate, benzoate, butyrate, citrate, fumarate, hypurate, propionate, succinate, valerate, alanine, arginine, aspartic acid, cysteine, L-phenylalanine, glutamine, lysine, methionine, ornithine, and serine.

#### 2.4.5. Chemotaxonomic Characterization

The strain S35^T^ was grown on medium 1 at 37 °C for 48 h and the fatty acids composition was determined following the protocol recommended by MIDI Microbial Identification System [49]. The fatty acids were determined by gas chromatography at the Spanish Type Culture Collection (CECT), Valencia, Spain.

## 3. Results and Discussion

### 3.1. Phylogenetic Analysis Based on 16S rRNA Gene Sequence Comparison

The 16S rRNA gene sequence comparative analysis of strain S35^T^ with respect to the type strains of *S. costicola* subsp. *costicola* CECT 4059^T^, *S. costicola* subsp. *alcaliphilus* DSM 16359^T^, *S. proteolyticus* DSM 19052^T^, *S. siamensis* JCM 14472^T^, *S. sharmensis* DSM 18182^T^, and *S. kushneri* AL184^T^ showed percentages of similarity of 99.2%, 99.4%, 97.7%, 97.7%, 97.9%, and 98.9%, respectively. These high percentages indicate that strain S35^T^ is a member of the genus *Salinivibrio*, but they are not conclusive to determinate if strain S35^T^ may constitute a novel species. The phylogenetic tree (Figure 1) based on the 16S rRNA gene sequences, constructed by the neighbor-joining algorithm, showed that strain S35^T^ clustered with the other species of the genus *Salinivibrio*. Topologies of phylogenetic trees inferred using the maximum likelihood and maximum parsimony were very similar to those of this tree.

### 3.2. Phylogenomic Comparative Analysis

Since the comparison of the 16S rRNA gene sequence does not allow us to determine in depth the phylogenetic relationships within the genus *Salinivibrio* [22], and in order to increase the resolution, we carried out a phylogenomic analysis based on the gene sequences obtained from the available genomes, whose characteristics are shown in Table S1, of strain S35^T^, *Salinivibrio costicola* subsp. *costicola* LMG 11651^T^, *Salinivibrio costicola* subsp. *alcaliphilus* DSM 16359^T^, *Salinivibrio proteolyticus* DSM 19052^T^, *Salinivibrio siamensis* JCM 14472^T^, *Salinivibrio sharmensis* DSM 18182^T^, and *Salinivibrio kushneri* AL184^T^.

**Figure 2:**
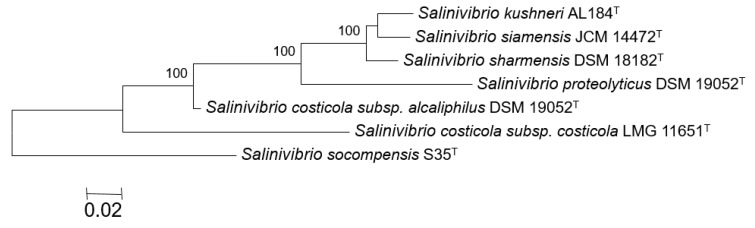
Approximately maximum likelihood tree based on the nucleotide sequences of the core genome (1265 genes) of strain S35^T^ and the type strains of the genus *Salinivibrio*. Bootstrap values over 70% are shown at the nodes. Bar, 0.02 substitutions per nucleotide position.

The core genome tree was based on 1265 common genes of the seven studied *Salinivibrio* strains. Figure 2 shows that strain S35^T^ constitutes a phylogroup different enough from the other type strains of *Salinivibrio* as to be considered as a new species. As shown in Figure 2, strain S35^T^ did not cluster together with any of the species and subspecies of *Salinivibrio*, being separated from all of them. Thus, we concluded that strain S35^T^ constitutes a new species of this genus.

### 3.3. ANI Values and In-Silico DNA–DNA Hybridization (GGDC)

Table 1 shows the ANIb, ANIm, and orthoANI percentages of strain S35^T^ with respect to the other species and subspecies of *Salinivibrio*. The current threshold value for delineating to bacterial species using the aforementioned indexes is 95%, meaning that if a result is above or equal to this value then the strains belong to the same species, but they constitute different species when this value is below 95% [44]. All ANI values obtained using the three different methods were below 95% between strain S35^T^ and the type strains of the species and subspecies of the genus *Salinivibrio*. Regarding GGCD, strain S35^T^ is beneath 70% threshold with respect to the type strains of the species of the genus *Salinivibrio* (Table 1). According to Kim et al. [50], GGDC percentages above or equal to 70% indicate that the strains can be assigned to the same species, and values under 70% indicate that the strains belong to different species.

### 3.4. Phenotypic Characterization

Colonies of strain S35^T^ showed cream-pink pigmentation and spherical shape, with a diameter lower than 3 mm. Cells were motile, Gram-stain-negative, curved rods, similar to those reported for other *Salinivibrio* species [14,15,16,17,18,19,20,21]. Strain S35^T^ was able to grow in a range of 3%–20% (w/v) NaCl, with an optimum at 7.0%–7.5% (w/v) NaCl (Figure S1). Strain S35^T^ can be considered as a moderately halophilic bacterium, as are the other species of *Salinivibrio* (Table 2). The pH allowing growth ranged from 6 to 10, with an optimum at pH 8.0 (Figure S2). These values are similar to those described for other *Salinivibrio* strains, except for *S. costicola* subsp. *alcaliphilus* and *S. sharmensis*, that had optimum pH at 9.0. The temperature range for growth was 10–42 °C with an optimal growth at 37 °C. Biochemical and nutritional characteristics of strain S35^T^ with respect to those of the species and subspecies of the genus *Salinivibrio* are given in Table 2. It is noticeable that strain S35^T^ is not able to hydrolyze any of the substrates tested, with the exception of DNA and gelatin. On the other hand, in contrast with the other species of *Salinivibrio,* strain S35^T^ is the only one that showed a positive result for the methyl red test. Finally, strain S35^T^ has predominance for the utilization of sugars instead of alcohols or organic acids as sole source of carbon and energy (Table 2).

### 3.5. Chemotaxonomic Characterization

The percentage of the major fatty acids composition, determined by gas chromatography, is shown in Table S2. Fatty acids C_16:0_ (20.8%), C_16:1_ *ω*7*c,* and C_16:1_ *ω*6*c* (summed feature 3; 26.2%), and C_18:1_ *ω*7*c* and/or C_18:1_ *ω*6*c* (summed feature 8; 14.4%) were predominant in strain S35^T^, similarly to other species of the genus *Salinivibrio*, or even of the genus *Vibrio*, both genera included in the family *Vibrionaceae* [51,52].

## 4. Conclusions

On the basis of the results of the polyphasic taxonomic analysis, it is concluded that strain S35^T^ should be considered as a novel species of the genus *Salinivibrio*, for which the name *Salinivibrio socompensis* sp. nov. is proposed. We enclose the taxonomic description of this new species.

**Description of *Salinivibrio socompensis* sp. nov.**

*Salinivibrio socompensis* (so.com.pen’sis L. masc. adj. *socompensis*, belonging to lake Socompa, Argentina).

Cells are Gram-stain-negative, motile, non-endospore-forming, curved rods. Colonies on SW7.5 medium are circular, with entire edges, convex, 2–3 mm diameter, and cream-pink-pigmented. Moderately halophilic, able to grow at 3%–20% (w/v) NaCl (optimally at 7.0–7.5% [w/v] NaCl); pH 6.0–10.0 (with an optimum at 8.0); and 10–42 °C (optimally at 37 °C). Facultatively anaerobic. Positive for catalase, oxidase, hydrolysis of gelatin and DNA, methyl red test, and nitrate and nitrite reduction. Negative for the hydrolysis of starch, casein, aesculin, and Tween 80; production of indole; Simmon’s citrate; Voges–Proskauer; H_2_S production; ornithine, arginine, and lysine decarboxylases; urease; and phenylalanine deaminase. Acid is produced from D-fructose, D-galactose, glycerol, D-glucose, lactose, D-trehalose, and sucrose, but not from D-arabinose, maltose, mannitol, or D-xylose. Amygdalin, cellobiose, D-glucose, maltose, raffinose, sucrose, dulcitol, glycerol, starch, citrate, propionate, succinate, or valerate are used as sole carbon and energy source, but not D-arabinose, D- fructose, D-galactose, lactose, D-mannose, melezitose, melibiose, D-trehalose, butanol, ethanol, mannitol, methanol, propranolol, sorbitol, xylitol, acetate, benzoate, butyrate, fumarate, or hypurate. The amino acids alanine, arginine, and ornithine are used as sole carbon, nitrogen, and energy source, but not aspartic acid, cysteine, glutamine, L-phenylalanine, lysine, methionine, or serine. The major fatty acids are C_16:0_, C_16:1_ *ω*7*c,* and C_16:1_ *ω*6*c*, and C_18:1_ *ω*7*c* and/or C_18:1_ *ω*6*c*. The DNA G + C content is 49.5 mol% (genome).

The type strain is S35^T^ (= CECT 9634^T^ = BNM 0535^T^), isolated from the volcanic saline lake Socompa, Argentina.

The GenBank/EMBL/DDBJ accession numbers of the 16S rRNA gene sequence and complete genome sequence of the type strain S35^T^ are HF953987 and AQOD00000000, respectively.

## Figures and Tables

**Table 1:** Average nucleotide identity (ANI), calculated using three different methods (ANIb, ANIm, and orthoANI) and *in silico* DNA–DNA hybridization (GDDC) values (%) for strain S35^T^ and the type strains of the species and subspecies of the genus *Salinivibrio*.

	ANIb	ANIm	orthoANI	GGDC
**Strains**	**Strain S35^T^**			
*S. costicola* subsp. *costicola* LMG 11651^T^	87.8	88.9	88.1	35
*S. costicola* subsp. *alcaliphilus* DSM 16359^T^	87.9	88.6	88.2	35
*S. proteolyticus* DSM 19052^T^	78.1	84.6	78.6	22
*S. sharmensis* DSM 18182^T^	80.4	84.5	80.8	23
*S. siamensis* JCM 14472^T^	80.6	84.6	80.9	24
*S. kushneri* AL184^T^	80.6	84.7	81.0	24

**Table 2:** Differential characteristics between strain S35^T^ and the type strains of the closely related species and subspecies of the genus *Salinivibrio*.

Characteristic	1	2	3	4	5	6	7
NaCl range (% w/v)	3–20	0.5–20 ^a^	2–25 ^b^	1–17 ^c^	1–22 ^d^	6–16 ^e^	2–20 ^f^
NaCl optimum (% w/v)	7–7.5	10 ^a^	10 ^b^	5 ^c^	10 ^d^	10 ^e^	7.5 ^f^
pH range	6–10	5–10 ^a^	7–10.5 ^b^	5–9.5 ^c^	5–9 ^d^	6–10 ^e^	5–10 ^f^
pH optimum	8	7.5 ^a^	9 ^b^	8 ^c^	8 ^d^	9 ^e^	7.2–7.4 ^f^
Temperature range (°C)	10–42	5–45 ^a^	10–40 ^b^	10–45 ^c^	10–47 ^d^	25–40 ^e^	17–49 ^f^
Temperature optimum (°C)	37	37 ^a^	30 ^b^	35 ^c^	37 ^d^	35 ^e^	37 ^f^
Hydrolysis of starch	−	+	−	+	+	+	+
Hydrolysis of Tween 80	−	+	−	+	−	−	−
Hydrolysis of casein	−	−	+	−	−	+	−
Hydrolysis of aesculin	−	+	+	−	−	+	−
Production of indole	−	−	−	−	+	−	−
Methyl red test	+	−	−	−	−	−	−
Nitrate and nitrite reduction	+	+	+	−	+	+	+
Arginine decarboxylase	−	+	+	−	+	+	−
Acid production from carbohydrates:							
D-fructose	+	+	+	−	+	+	+
D-galactose	+	−	+	−	−	−	−
Lactose	+	−	+	−	−	−	−
Maltose	−	−	+	+	+	+	−
Mannitol	−	+	+	+	+	+	−
Sucrose	+	+	+	+	+	−	+
D-xylose	−	+	+	−	−	−	−
Utilization as sole carbon and energy source of the:							
Amygdalin	+	−	−	+	+	+	−
D-arabinose	−	−	−	+	+	−	−
Cellobiose	+	−	−	+	+	+	−
D-fructose	−	−	−	−	+	+	−
D-glucose	+	−	−	+	+	+	+
Maltose	+	−	−	+	+	+	+
D-mannose	−	+	+	+	+	+	+
Melibiose	−	−	+	−	−	−	−
Sucrose	+	−	−	+	+	+	+
Starch	+	−	−	+	+	+	−
D-trehalose	−	−	−	+	+	−	−
D-xylose	+	−	−	+	+	+	−
Butanol	−	+	+	−	−	−	−
Ethanol	−	−	+	+	+	+	+
Glycerol	+	−	−	+	+	+	+
Mannitol	−	+	+	+	+	+	+
Methanol	−	−	+	−	+	−	+
Propranolol	−	−	+	−	+	+	+
Sorbitol	−	−	−	+	−	−	−
Xylitol	−	−	−	−	+	+	−
Benzoate	−	+	−	+	+	+	−
Butyrate	−	−	−	+	+	+	+
Citrate	+	−	−	+	−	+	−
Fumarate	−	−	−	+	+	+	−
Hypurate	−	−	−	+	+	+	+
Succinate	+	−	−	+	+	−	+
Valerate	+	-	-	-	+	+	+
Utilization as sole carbon, nitrogen and energy source of:							
Alanine	+	-	-	+	+	+	+
Arginine	+	-	-	+	+	+	+
Aspartic acid	-	-	-	+	+	-	+
Cysteine	-	-	-	+	+	-	+
Glutamine	-	-	-	+	+	-	-
L-phenylalanine	-	-	-	+	+	+	+
Ornithine	+	-	-	+	+	+	+
Serine	-	-	-	-	+	-	-

1. Strain S35^T^; 2. *S. costicola* subsp. *costicola* DSM 11403^T^; 3. *S*. *costicola* subsp. *alcaliphilus* DSM 16359^T^; 4. *S. proteolyticus* DSM 19052^T^; 5. *S. siamensis* JCM 14472^T^; 6. *S. sharmensis* DSM 18182^T^; 7. *S. kushneri* AL184^T^. All strains were positive for catalase, oxidase, and hydrolysis of DNA and gelatine, and negative for Voges–Proskauer, Simmon’s citrate, H_2_S production, ornithine decarboxylase, lysine decarboxylase, urease, and phenylalanine deaminase. Acid was produced from glycerol, D-glucose, and D-trehalose but not from D-arabinose. All strains were positive for raffinose, dulcitol, and propionate as sole carbon and energy source, and negative for D-galactose, lactose, melezitose, and acetate, along with lysine and methionine as sole carbon, nitrogen, and energy source. ^a^ Mellado et al. [14]. ^b^ Romano et al. [16]. ^c^ Amoozegar et al. [17]. ^d^ Chamroensaksri et al. [18]. ^e^ Romano et al. [19]. ^f^ López-Hermoso et al. [20].

## Supplementary Materials

The following are available online at https://www.mdpi.com/2076-2607/7/8/241/s1. Table S1. Genomes of the type strains of species of the genus Salinivibrio available in GenBank database used in this study, including their basic statistical information. Table S2. Cellular fatty acids composition (%) of strain S35T and the type strain of species and subspecies of the genus Salinivibrio. Figure S1. Growth curve for strain S35T at different salt concentrations. Culture media had the same composition than the medium used for routinely growth at 3, 6, 7, 7.5, 8, 9, 10, 15 and 20% (w/v) total salt, respectively. Figure S2. Growth curve for strain S35T at different pH values. Culture media had the same composition than the medium used for routinely growth and the pH was adjusted to 5, 6, 7, 7.5, 8, 9 and 10, respectively.
