# Supplementary material for: Characterization of Salinivibrio socompensis sp. nov., A New Halophilic Bacterium Isolated from the High-Altitude Hypersaline Lake Socompa, Argentina
Source: Microorganisms. 2019 Aug 5;7(8):241. doi: 10.3390/microorganisms7080241 (PMC6723482; doi:10.3390/microorganisms7080241)
Supplement: Supplementary file 1 [file microorganisms-07-00241-s001.pdf]

Table S1. Genomes of the type strains of species of the genus *Salinivibrio* available in GenBank database used in this study, including their basic statistical information.

Strain designation	Assembly no.	Completeness level	Size (Mb)	G + C (mol%)	Scaffolds	CDS
Salinivibrio costicola subsp. *costicola* LMG 11651^T	GCA_000565345.1	Contig	3.38	49.3	202	2332
Salinivibrio costicola subsp. *alcaliphilus* DSM 16359^T	GCA_001996185.1	Contig	3.38	49.3	248	2949
Salinivibrio kushneri AL184^T	CP040021, CP040022	Complete	3.44	50.7	1	3055
Salinivibrio proteolyticus DSM 19052^T	GCA_001996165.1	Contig	3.6	49.8	51	3234
Salinivibrio sharmensis DSM 18182^T	GCA_001995985.1	Contig	3.33	50.3	40	2944
Salinivibrio siamensis JCM 14472^T	GCA_001996005.1	Contig	3.44	50.4	61	3025
Salinivibrio socompensis S35^T	GCA_000513715.1	Contig	3.41	49.5	270	2311

Table S2. Cellular fatty acids composition (%) of strain S35^T and the type strain of species and subspecies of the genus *Salinivibrio*.

Fatty acids (%)	1	2[a]	3[a]	4[a]	5[a]	6[a]	7
$C_{12:0}$	4.9	7.6	10.5	6.7	12.7	3.1	3.5
$C_{12:0}$ 3OH	4.3	-	-	-	-	-	2.4
$C_{14:0}$	1.0	3.7	2.8	7.1	9.8	4.7	2.1
Summed feature 2*	4.4	-	-	-	-	-	2.7
$C_{16:0}$	20.8	19.8	27.8	21.6	29.2	54.0	23.8
$C_{16:1}$	N.D.	29.6	37.3	24.0	12.8	26.0	N.D.
$C_{16:1}$ $\omega 9c$	3.0	-	-	-	-	-	N.D.
Summed feature 3*	26.2	-	-	-	-	-	43.8
$C_{17:0}$ cyclo	8.0	-	-	-	-	-	N.D.
$C_{18:0}$	4.2	2.7	7.9	3.1	1.9	1.8	2.6
$C_{18:1}$	N.D.	31.2	12.7	33.7	14.5	9.1	N.D.
$C_{18:1}$ $\omega 9c$	2.9	-	-	-	-	-	1.1
Summed feature 8*	14.4	-	-	-	-	-	13.1

1. Strain S35^T; 2. *S. costicola* subsp. *costicola* DSM 11403^T; 3. *S. costicola* subsp. *alcaliphilus* DSM 16359^T; 4. *S. proteolyticus* DSM 19052^T; 5. *S. siamensis* JCM 14472^T; 6. *S. sharmensis* DSM 18182^T; 7. *S. kushneri* AL184^T. [a] Romano et al. [19].Values lower than 1.0% are not shown. N.D., Not detected. -, Data not available.

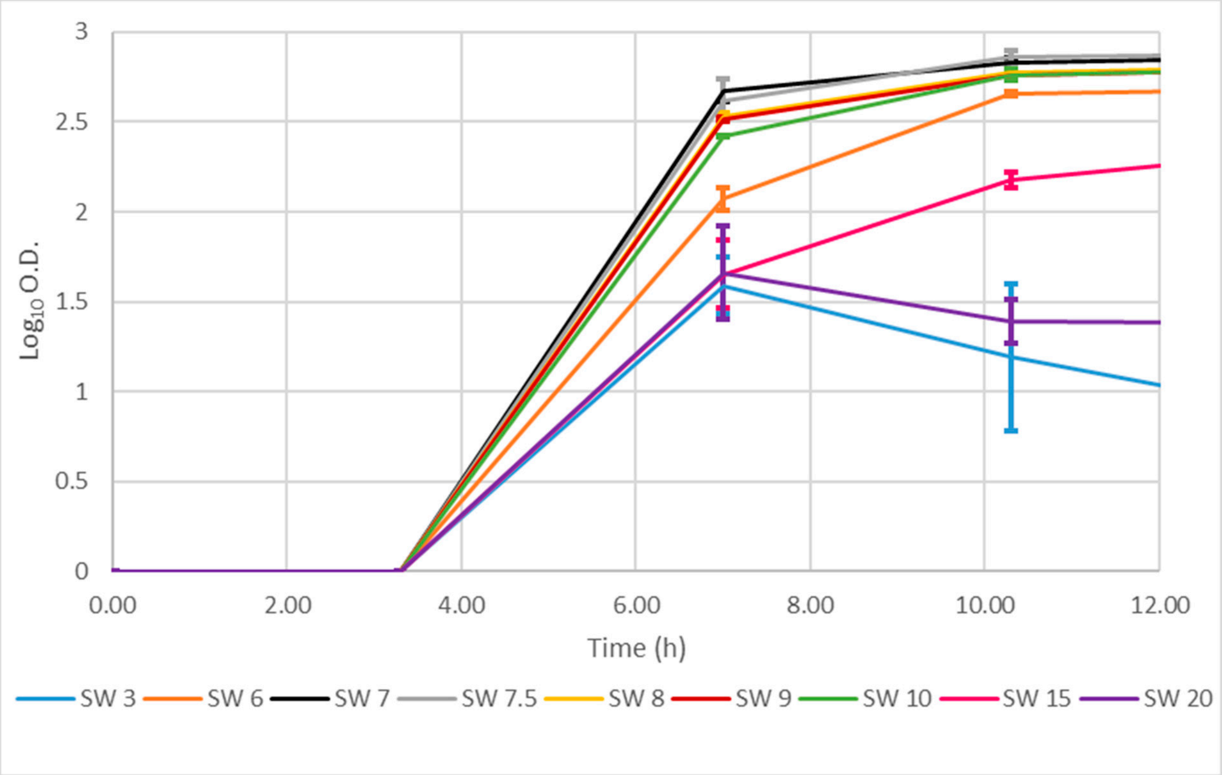

*Summed features refer to those fatty acids with imperfect peak separation by gas chromatography. Summed feature 2 comprised $C_{14:0}$ 3OH and $C_{16:1}$ iso, summed feature 3 comprised $C_{16:1}$ *ω7c* and $C_{16:1}$ *ω6c* and summed feature 8 comprised $C_{18:1}$ *ω7c* and/or $C_{18:1}$ *ω6c*.

Figure S1. Growth curve for strain S35^T at different salt concentrations. Culture media had the same composition than the medium used for routinely growth at 3, 6, 7, 7.5, 8, 9, 10, 15 and 20% (w/v) total salt, respectively.

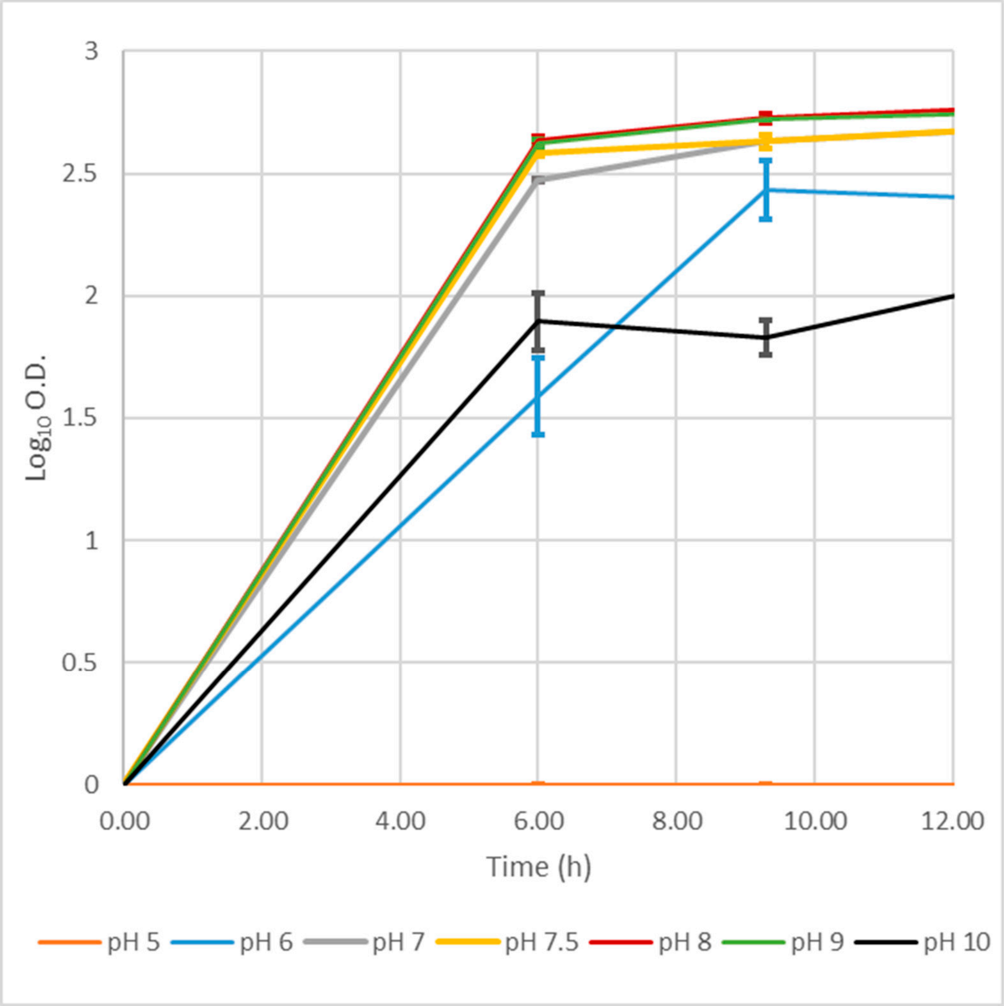

Figure S2. Growth curve for strain S35^T at different pH values. Culture media had the same composition than the medium used for routinely growth and the pH was adjusted to 5, 6, 7, 7.5, 8, 9 and 10, respectively.
